# Bladder ultrasonography in the assessment of cauda equina syndrome in the emergency department: a literature review

**DOI:** 10.1308/rcsann.2022.0168

**Published:** 2023-04-13

**Authors:** NV Todd

**Affiliations:** Nuffield Health Newcastle-upon-Tyne Hospital, UK

**Keywords:** Cauda equina syndrome, Perianal sensation, Anal tone, Bladder ultrasonography, Magnetic resonance imaging

## Abstract

For cauda equina syndrome (CES), current clinical assessment in the emergency department usually involves perianal sensation (PAS) and anal tone (AT). Neither reliably predict magnetic resonance imaging (MRI) demonstrating a large central disc prolapse (MRI+). Other clinical examination findings increase the probability of MRI+. Other tests of sacral nerve root function include anal squeeze (AS) and the bulbocavernosus reflex (BCR). If BCR, PAS and AT, and AS are combined and they are all normal, CES can be excluded in almost all cases. Portable bladder ultrasonography is now commonly used to assess bladder function, particularly in measuring the post-void residual urinary volume (PVR). PVR is deemed normal at <50ml. If the PVR is <200ml and there are no objective signs, MRI+ is rare. If the PVR is >200ml, MRI+ is found in 43% of cases.

The combined assessment of PAS, AT and AS (and BCR in selected cases) and PVR increases the specificity and sensitivity of a clinical diagnosis of CES (i.e. maximising MRI+ and minimising MRI−). Recommendations for when to perform MRI are made.

## Introduction

Cauda equina syndrome (CES) is an emergency spinal pathology that commonly presents to doctors in the emergency department. If a prompt diagnosis of CES is not achieved, there can be severe, irreversible loss of bladder, bowel and sexual function, which often has a devastating impact on the patient and can lead to expensive litigation.^1^

There is no single symptom or clinical sign that predicts CES, and even combinations of symptoms and signs have a high predictive value only when CES is advanced and frequently irreversible.^2–6^ Portable bladder ultrasonography (BUS) is available in most emergency departments and is increasingly used in the diagnosis of CES as well as in determining when potential CES patients should be referred for magnetic resonance imaging (MRI).^7,8^ This systematic review considered the role of BUS in the emergency assessment of potential CES patients, the implications of urinary volumes and other clinical findings in the diagnosis of CES, and whether/when MRI should be performed.

## Methods

A literature search was performed on the PubMed^®^ and MEDLINE^®^ databases, in accordance with the PRISMA (Preferred Reporting Items for Systematic reviews and Meta-Analyses) guidelines,^9^ employing the search terms “cauda equina syndrome” AND “bladder ultrasound”.

## Results

The literature search returned 46 abstracts. Seven full papers were selected for review. The remaining 39 abstracts did not report useful clinical information. The 7 full papers reviewed yielded a further 11 papers and so 18 papers were reviewed in total.

The absence of physical signs can exclude CES. In a single centre review of 142 suspected CES (sCES) cases, normal perianal sensation (PAS), anal tone (AT) and bulbocavernosus reflex (BCR) were associated with negative MRI (MRI−) in 100% of cases.^10^

Deyo *et al* found that urinary retention (volume not specified) had a 90% sensitivity and a 99% specificity for positive MRI (MRI+).^11^ Furthermore, the absence of urinary retention excluded CES in 99.9% of cases.

Domen *et al* retrospectively reviewed 58 patients undergoing urgent lumbar MRI for sCES.^12^ Eight were MRI+ and all eight had emergency surgery within 24 hours (mean: 13.2 hours, standard deviation: 4.7 hours). Preoperative post-micturition ultrasonography was performed in six cases and all had a post-void residual urinary volume (PVR) of >500ml. There was reduced sensation of micturition in 25%, clinical urinary retention in 87.5%, urinary incontinence in 37.5% and faecal incontinence also in 37.5% of cases. Objective signs included reduced PAS and reduced anal sphincter (AS) reflex in 37.5% (each) and reduced AT in 25% of cases. The odds ratio for MRI+ was 4.0 where PVR was >500ml, 15.5 with a PVR of >1,000ml, and 48 with a PVR of >500ml and two of three clinical features (bilateral sciatica, urinary retention or faecal incontinence).

Hoeritzauer *et al* retrospectively reviewed 276 patients with sCES.^5^ Over a quarter (78/276, 28%) were MRI+ and of these, 86% (67/78) had disc prolapses. PVRs were measured in 65 cases. Among the MRI+ cases, the PVR was <100ml in 33%, 100–500ml in 47% and >500ml in 20% of patients. For a PVR of >100ml, the sensitivity of a positive MRI was 67% (i.e. 67% of the MRIs were normal) with a specificity of 52% (i.e. 52% of the MRIs were positive).

Venkatesan *et al* retrospectively reviewed 92 patients with sCES.^7^ The pre and post-void BUS results for all cases were 470ml and 248ml respectively; for MRI+ (17/92, 18%), they were 672ml and 466ml, and for MRI− (75/92, 82%), they were 424ml and 199ml. The odds ratio for a positive MRI was 20.7 with PVR ≥200ml. The clinical signs in MRI+ cases were reduced PAS (82%), reduced/absent AT (53%) and absent voluntary anal contraction (VAC) (29%). In MRI− cases, reduced PAS on examination was present in 56% and reduced/absent AT was present in 37% of patients. A PVR of ≥200ml had a probability of MRI+ of 43% (sensitivity 94%, specificity 72%). Venkatesan *et al* concluded that if there was normal PAS and a PVR of <200ml, then MRI could be deferred until the next day. One (6%) of the 17 patients with positive MRI had bilaterally reduced PAS with a PVR of <200ml.

Katzouraki *et al* performed a prospective study of 260 patients with sCES.^8^ MRI+ was found in 34 (13%), all of whom underwent emergency surgery. The average degree of canal occlusion in the MRI+ cases was 76% (95% confidence interval: 72–81%). The clinical features were urinary incontinence (97%), saddle anaesthesia (73%), reduced PAS (63%) and reduced/absent AT (33%). The PVR was ≥200ml in 94% of MRI+ cases (with 6% ≤200ml). Of the four cases with a PVR of <200ml, two had normal PAS and AT, two had unilateral reduction of PAS, one had reduced AT and in one, AT was normal; all four underwent emergency surgery. PVR >200ml had 94% sensitivity in predicting MRI+ (specificity 67%, positive predictive value 30%, negative predictive value 99%). Katzouraki *et al* concluded that urgent MRI is not needed if the PVR is <200ml and there is normal PAS and VAC. MRI can therefore be deferred to normal working hours or be performed on a routine basis. None of the 34 cases (13%) where the MRI was deferred had positive MRI and none developed CES.

Kalidindi *et al* assessed 249 sCES cases, with formal urodynamic studies (UDS) being performed in the majority.^13^ All patients had low back pain, unilateral or bilateral radiculopathy, bladder symptoms or signs of CES and were MRI+. There was urinary frequency or urgency in 115 (46%), urinary retention in 87 (35%) and incontinence in 47 (19%). Uroflowmetry (UFM) and PVR were performed. In 34 cases (14%), they were normal and CES was excluded. Of the remaining 215 patients, 211 agreed to undergo invasive UDS. Thirty-three (16%) of the 211 patients had a contractile bladder and CES was excluded. Eighty-five (40%) had a hypocontractile bladder; there was reduced PAS, VAC or BCR in 66 (78%) of these and CES was diagnosed. Of the 19 cases with normal PAS, VAC and BCR, the most common causes of abnormal UFM/PVR were pain and medication. Ten patients were treated conservatively and none deteriorated. Of the 66 patients with hypocontractile bladders with reduced PAS, VAC and/or BCR, 54 (82%) had a high PVR and all underwent emergency surgery. In the 12 individuals with hypocontractile bladders and normal PAS, VAC and/or BCR, 9 had surgery for other reasons. Among the three cases treated conservatively, one patient developed abnormal signs and underwent surgery. Ninety-three (44%) of the 211 patients who agreed to UDS had an acontractile bladder. Of these, 86 (92%) had abnormal signs and underwent surgery. Seven (8%) had pain or drug induced bladder problems. (Of four treated conservatively, none deteriorated.) Overall, only 141 (58%) of the 245 patients in the study by Kalidindi *et al* (excluding 4 drop-outs) had neurogenic bladder symptoms from CES and underwent emergency surgery. Sixty-seven patients (27%) had non-neurological causes of bladder symptoms (including prostatic hypertrophy, drugs, pain, detrusor overactivity or stress incontinence) and were treated conservatively. High PVRs can be found in hypocontractile bladders of non-neurogenic origin and low PVRs can be found in neurogenic bladders if the patient passes urine with abdominal straining. Kalidindi *et al* concluded that PVR alone was not a sensitive test for CES.

## Discussion

CES is a constellation of symptoms and signs; not all will be present at the time of diagnosis. Typically, there is low back pain, often severe. There can be unilateral or bilateral radicular pain. There may also be lower limb numbness, paraesthesia and/or weakness. There is usually bladder, bowel and/or sexual dysfunction as well as subjective alteration of PAS. Objective signs are generally present as well as symptoms. The clinical symptoms and signs of CES do not reliably correlate with cauda equina compression on MRI and clinical assessment alone cannot reliably diagnose or exclude MRI-proven cauda equina compression.^2–6^ Symptoms suggestive of CES are very common in MRI− cases: bladder symptoms are present in 67% and bilateral sciatica in 20%.^5^ Up to 70% of MRI− cases will have reduced PAS and up to 51% will have reduced AT on physical examination.^14^

Failure to diagnose CES is frequently associated with long-term, severe neurological and functional disability, and there is often litigation. In order to avoid a misdiagnosis of CES, large numbers of MRI scans are performed that are negative and typically, less than 20% of MRI scans are positive.^7,8,15^ In the emergency department, two signs are usually assessed: PAS and AT. The clinical assessment of AT is inaccurate;^16^ in a model simulating AT, only 64% of AT assessments were correct.^17^ PAS and AT are not the most sensitive signs of sacral nerve root injury. The assessment of anal squeeze (AS) may be more accurate than AT.^17^

BCR is rarely tested in CES but an absent BCR has a high correlation with MRI+.^9^ The reflex is tested by squeezing the glans penis or clitoris (or tugging on a bladder catheter) and observing contraction of the anal sphincter, which is the normal response. No anal sphincter contraction is evidence of sacral nerve root injury. BCR is currently not used widely and it is a difficult examination in women in the lateral position. The high correlation of a lack of BCR and MRI+ may be due to an absent BCR being more likely in severe CES. Assessment of BCR is controversial and is unlikely to be widely adopted. Some argue that because no single sign predicts MRI+ accurately in sCES cases, BCR examination cannot be relied on. However, the accuracy of clinical diagnosis of CES increases if several signs of sacral nerve root injury are assessed.^18^

Portable BUS is now commonly used to assess patients with sCES. A probe is placed over the suprapubic area of the abdomen with the patient prone, and bladder images are recorded in sagittal and transverse planes. The ultrasonography machine automatically calculates urinary volumes.

BUS is unsuitable for patients with severe abdominal scars, uterine prolapse, pregnancy or abdominal ascites and the technique is operator-dependant.^19^ BUS can tell us about pre and post-void bladder volumes. Pre-void BUS quantifies the degree of bladder distention/retention. Post-void imaging tells us about the extent to which the bladder is emptying to completion. If there is urinary retention, the patient should be asked whether they have a normal sensation of bladder fullness. Painless retention of a large urinary volume suggests an insensate bladder, which is commonly found in CES. Nevertheless, in non-CES cases of lumbar disc disease, there is reduced bladder sensation in 50% of cases, of whom 25% strain to void.^20^ In patients with severe CES, some sensation of bladder fullness can be found in over 95% of patients^21^ although the sensation of bladder fullness is not normal. Patients should be asked whether they have a normal sensation of bladder fullness rather than whether there is a complete absence of sensation.

The PVR is the residual volume of urine in the bladder post-micturition. In young adults, a PVR of <50ml is deemed normal while in the older population, 50–100ml is considered normal.^22,23^ A high PVR is evidence of incomplete bladder emptying but not all of these patients have neurogenic bladder dysfunction.^13^ When detailed urological assessment (UFM, PVR and UDS) was performed in patients with positive symptoms and signs of CES and MRI+, 16% had a normal contractile bladder on UDS, 40% had a hypocontractile bladder (with 22% of these having no objective neurological deficit [i.e. there was no neurological cause for the hypocontractile bladder]) and 44% had an acontractile bladder (with 7.5% of these having no neurological cause for bladder dysfunction).^13^

The higher the PVR, the greater the probability of the patient being CES+ (i.e. having both clinical and radiological evidence of CES requiring emergency surgery).^5,7,8^ The Nottingham spinal unit has suggested a cut-off PVR of 200ml^7,8^ and as long as there are no abnormal neurological signs (normal PAS and VAC), emergency MRI is not required and the imaging can be deferred. It is important to note the qualification of normal PAS and VAC. A PVR of <200ml does not exclude symptomatic CES and can be found in up to 20% of CES+ cases.^11^ However, provided PAS and VAC are normal, a PVR of <200ml indicates that the risk of CES is minimal.^8^

In the two largest studies on CES, 3 (6%) of the 51 patients who were MRI+ had a PVR of <200ml (with positive signs).^7,8^ A recent study of 50 medicolegal cases found that in MRI+ patients, 50% had a PVR of <200ml.^22^ The author is aware of a number of clinical and medicolegal cases where doctors have assumed that a PVR of <200ml means that there is no risk of CES despite the presence of positive physical signs, which were ignored.^22^ It is probably sensible to regard the PVR as a continuous variable. A PVR of <200ml indicates a low probability of the patient being MRI+ whereas a PVR of >200ml has a probability of 43%.^7^ The PVR should be considered in conjunction with physical signs. The greatest probability of CES+ is found in patients with a higher PVR and positive signs of CES.^7,8,12^

The subclassification of CES has recently been reconsidered and expanded.^24^ The new subclassification is set out in Table 1 and it is hoped that this will increase the recognition of early CES, where good outcomes are likely to be achieved. Most patients who are capable of passing urine (to achieve a PVR) are incomplete CES (CESI) patients; there will be a small number of exceptions where patients pass urine by straining.^13^ CESI patients are in a favourable group for which outcomes are typically better than for patients with CES with neurogenic retention of urine (CESR). Patients with a very high PVR (typically >1,000ml) will most commonly have CESR.

**Table 1:** Subclassification of cauda equina syndrome (CES)^24^

CESS (CES suspected or suspicious)	Bilateral radiculopathy, no CES, increased risk of CES
CESE (CES early)	Symptom-only CES, symptoms of perineal sensory impairment and/or a change in bladder function, often no objective signs
CESI (CES incomplete)	Symptoms and objective signs of CES with voluntary control of micturition (executive bladder control)
CESR (CES with neurogenic retention of urine)	Paralysed insensate bladder with overflow incontinence (loss of executive bladder control)

MRI is a finite resource, particularly in district general hospitals, where the primary diagnosis of CES is usually made. It is good practice to minimise out-of-hours MRI, provided this has no or minimal effect on patient safety. The ideal, of course, is to perform MRI for all patients who will be MRI+ and for the lowest possible number of MRI− patients but significant rates of negative MRIs are the price of minimising false negative CES. The presence or absence of objective signs of CES and the PVR can be used to determine the urgency of MRI in the emergency department.^23^ Symptom-only CES is uncommon. These are patients who have symptoms strongly suggesting CES but no abnormal signs. The probability of such a patient having a positive MRI is low. Such cases should be discussed with the local spinal service.

## Conclusions

In the emergency department, standard practice should be to take a history of potential symptoms of CES, and then to perform a detailed neurological examination including PAS, AT and AS followed by BUS to determine the PVR. In the author’s opinion, if all four are normal, the patient can be discharged with a cauda equina warning card.

Conversely, if there is any positive sign (PAS, AT or AS), then CES cannot be excluded even if the PVR is <200ml and emergency MRI is required. If the PVR is >200ml, emergency MRI is required regardless of signs (although most MRI+ cases will have signs). If there are no signs but the PVR is not normal (i.e. 50–200ml), there will be a few CES+ cases and MRI should ideally be performed within 24 hours. PVR is insensitive as a stand-alone test of bladder function.^13^ There are non-neurological causes of abnormal PVR and for cases with a high PVR but no signs of CES, there should be formal UDS as soon as practicable.
